# ArTisaN trial protocol: a single Centre, open-label, phase II trial of the safety and efficacy of TheraSphere selective internal radiation therapy (SIRT) in the treatment of inoperable metastatic (liver) neuroendocrine neoplasia (NENs)

**DOI:** 10.1186/s12885-022-09859-9

**Published:** 2022-07-20

**Authors:** Rohini Sharma, Susanna Slater, Joanne Evans, Maria Martinez, Caroline Ward, Hooshang Izadi, Florian Wernig, Rob Thomas

**Affiliations:** 1 Division of Surgery and Cancer, Hammersmith Hospital, Imperial College London, Du Cane Road, London, W12 0NN UK; 2 Department of Oncology, Hammersmith Hospital, Imperial College NHS Healthcare Trust, Du Cane Road, London, W12 0HS UK; 3 Department of Mechanical Engineering and Mathematical Sciences, Oxford Brookes University, Oxford, UK; 4 Department of Endocrinology, Hammersmith Hospital, Imperial College NHS Healthcare Trust, Du Cane Road, London, W12 0HS UK; 5 Department of Interventional Radiology, Hammersmith Hospital, Imperial College NHS Healthcare Trust, Du Cane Road, London, W12 0HS UK

**Keywords:** Neuroendocrine neoplasia, Selective internal radiotherapy, Response, Safety, Quality of life

## Abstract

**Background:**

Neuroendocrine neoplasias (NENs) are a rare type of malignancy that arise from the cells of the neuroendocrine system. Most patients present with advanced, unresectable disease, typically with metastases to the liver. The presence of liver metastases dictates prognosis and there has been a number of studies investigating therapies that reduce the burden of liver disease. Selective Internal Radiation Therapy (SIRT) allows the delivery of targeted high dose radiation directly to tumours, with relative sparing of the surrounding liver tissue. Here, we describe the design and rationale of ArtTisaN, a phase II study to assess efficacy and tolerability of SIRT using TheraSpheres for the management of liver metastases secondary to NENs.

**Methods:**

Twenty-four eligible participants will be recruited to receive SIRT with TheraSpheres. The primary objective is to determine the objective response rate to treatment, defined as the rate of best overall response in the treated liver volume. In addition, total hepatic response and overall response will be assessed according to RECIST 1.1. The second co-primary objective is to determine the incidence of adverse and serious adverse device events. The secondary objectives are progression free survival, overall survival and quality of life. Additional exploratory objectives include investigation of circulating biomarkers of response and identification of a radiomic signature of response.

**Discussion:**

This trial will provide prospective evidence on the efficacy of SIRT using TheraSpheres for the management of liver metastases.

**Trial registration:**

NCT04362436.

**Supplementary Information:**

The online version contains supplementary material available at 10.1186/s12885-022-09859-9.

## Background

Neuroendocrine neoplasias (NENs) are a rare type of cancer that arise from the cells of the neuroendocrine system, most commonly within the gastroenteropancreatic and bronchopulmonary system [3]. The majority of NENs present with vague symptomology and as a result, 20–50% of patients present with metastatic disease, the commonest site being the liver [14]. The presence of metastatic disease per se confers a 5-fold increase in mortality risk [3], with hepatic metastases being a powerful prognostic predictor of survival [10, 20]. Therefore, there has been interest in investigating liver directed therapies in an effort to improve clinical outcome.

Current European Neuroendocrine Tumour Society (ENETs) guidelines suggest a number of first line systemic therapies for patients with either diffuse liver metastases or liver metastases not amendable to surgery [14]. Somatostatin analogues (SSAs), octreotide and lanreotide, are used as first line systemic therapy for unresectable grade 1 and 2 NENs [15]. Whilst initially introduced to the therapeutic armamentarium to provide symptomatic relief for secretory symptoms related to NENs, SSAs have been shown in two large randomised trials to improve progression free survival (PFS) [2, 18]. In the PROMID study, octreotide LAR was shown to significantly slow tumour progression and reported a PFS of 14.3 months compared to placebo (PFS 6 months), in patients with unresectable, metastatic, low grade, well differentiated NENs of mid-gut origin [18]. Of note, the anti-proliferative effect was greatest in patients with a low hepatic tumour burden (< 10%). Similar findings were reported with the use of lanreotide autogel in the CLARINET study [2].

Other systemic agents include everolimus [16], sunitinib [17] and chemotherapy all of which result in a low proportion of objective response [14]. Recently the use of peptide related radiation therapy (PRRT) has gained traction since the publication of the NETTER-1 study [21] which reported a significantly prolonged PFS in patients randomised to receive ^177^Lu-DOTATATE in combination with octreotide compared with octreotide alone (28.4 months vs 8.4 months, *p* < 0.001). However the reported objective response rate was low, 18% of patients achieved a response at 3 months following ^177^Lu- DOTATATE therapy [21]. A subgroup analysis of the NETTER-1 data investigated the impact of liver disease on treatment outcomes and illustrated that patients with target liver lesions > 30 mm derived less clinical benefit from ^177^Lu- DOTATATE compared to those with smaller lesions [22]. There is a need therefore for more effective liver directed therapy.

Liver metastases from NENs derive their blood supply predominantly from the hepatic artery enabling the delivery of targeted therapy directly to the tumour with minimal damage to the surrounding liver parenchyma. Transarterial embolization with (TACE) or without (TAE) chemotherapy allows the selective embolization of the main feeding vessel to the tumour resulting in an acute tumoural ischaemia and necrosis. A recent meta-analysis reported a median PFS of 22.1 months for TAE and 19.2 months for TACE [12]. Both procedures are generally safe when performed at experienced centres, however post-embolization syndrome (consisting of right upper quadrant pain, fever, nausea and vomiting) is not uncommon, occurring in 41% of patients following TAE and 61% after TACE according to retrospective data of 30 patients [7]. Whilst both TACE and TAE are recommended by ENETs guidelines (levele II evidence) [14], for the management of liver metastases is there is a lack of randomised, prospective studies.

SIRT allows the delivery of yttrium-90 (^90^Y) or holmium-166 (^166^Ho) labelled microspheres to the tumour via the hepatic artery. SIRT results in both hepatic arterial embolization and the targeted delivery of high dose radiation providing safe and effective locoregional therapy [23]. Whilst there are no randomised trials comparing SIRT to TAE/TACE, published work illustrates that SIRT is associated with less toxicity, shorter-in hospital stay and fewer treatment sessions [4, 6]. A recent analysis of 27 studies reported a collective response of 51% and disease control rate of 88% with a median overall survival (OS) of 32 month s[8]. A further analysis which reviewed 11 studies involving 870 patients reported an average disease control rate of 86 %[11]. The median OS was 28 months, with patients with pancreatic primaries deriving less benefit. Response within the liver correlated with survival outcomes.

TheraSpheres (Boston Scientific) are ^90^Y coated glass microspheres, with a sphere diameter ranging from 20 to 30 μm, achieving a microsphere concentration 22,000–73,000 microspheres per milligram. ^90^Y is a pure beta-emitter, which decays to stable zirconium-90 with a physical half-life of 64.2 hours. The average energy of the beta-emissions from ^90^Y is 0.9367 MeV with mean tissue penetration of 2.5 mm. As a result, a high dose of radiation is delivered to the tumour, while minimal dose is delivered to the surrounding liver parenchyma, thus limiting toxicity to the patient. TheraSpheres have been investigated for the management of a number of cancer types including cholangiocarcinoma and hepatocellular cancer (HCC) but not NENs. The Legacy study investigated the role of TheraSpheres for the management of locoregional HCC and confirmed high response rates and comparable survival data compared to other locoregional therapies [19]. In unresectable intrahepatic cholangiocarcinoma, the interim results of a phase II study combining TheraSpheres with chemotherapy suggest a 93% response rate with a median overall survival of 22 months suggesting good activity [5].

Whilst there are a large number of retrospective case series highlighting the utility of SIRT for the management of liver metastases secondary to NENs, prospective studies are lacking. The overall aim of the study is to assess the efficacy and safety of TheraSpheres for selective internal radiation therapy in patients with liver metastases secondary to NENs. In particular quality of life (QoL) assessments will be conducted, a key consideration in this otherwise palliative patient population. Moreover, we will investigate both radiomics and circulating biomarkers of response a key consideration given the number of therapies that are available for patients and the lack of available predictors of response.

## Methods and analysis

### Trial objectives

The primary objective is to determine the objective response rate (ORR) to treatment with TheraSpheres, defined as the rate of best overall response (which includes complete responses and partial responses) in the treated liver volume as determined by RECIST 1.1. In addition, total hepatic response and overall response will be assessed according to RECIST 1.1. The second co-primary objective is to determine the incidence of adverse and serious adverse device events (ADEs/SADEs) overall and by severity, graded by the National Cancer Institute - Common Toxicity Criteria (NCI-CTC v5.0).

The secondary objectives are to determine PFS defined as the time from the date of treatment to the date of the first documentation of disease progression and determined by RECIST 1.1 and OS defined as the time from the date of randomisation to the date of death from any cause (or censored at the date of the last contact). Quality of life (QoL) will be determined using the European Organisation for Research and Treatment of Cancer (EORTC) questionnaires QLQ-C30 and QLQ-GI.NET21 at baseline and every 3 months until disease progression. Exploratory endpoints include the assessment of radiomics in the prediction of treatment response determined by computed tomography (CT) scans of the liver at baseline, 12 weeks post SIRT and at disease progression. Bloods will also be taken for circulating biomarkers at baseline, 12 weeks post SIRT and at disease progression.

### Eligibility criteria

Participants will be recruited from Hammersmith Hospital, Imperial College Healthcare NHS Trust and from the London Clinic. Patients will be provided with a verbal and written explanation of the trial and given the opportunity to discuss all aspects of the trial with both the clinician and research nurse. Patients who provide written consent to participate in the trial after at least 24 hours of consideration will be registered and therefore eligible to proceed to further assessment.

To be eligible patients must have inoperable liver dominant metastatic disease. Patients will be required to have histologic proven NENs and have progressed through at least one line of therapy. Metastases within the liver must occupy > 25% but be less than < 60% of the normal hepatic parenchyma. Detailed inclusion and exclusion criteria are outlined in Table 1. A total of 24 patients who fulfil the criteria and progress through the pre-SIRT investigations successfully will go on to receive TheraSphere SIRT.

**Table 1:** ArTisaN inclusion and exclusion criteria

ARTISAN inclusion and exclusion criteria	
Inclusion Criteria	Exclusion Criteria
1. Histologically confirmed neuroendocrine tumour, with documented grade. 2. > 18 years of age 3. Patients may be on SSAs concurrently. 4. Patients must have had at least one previous line of therapy 5. Unresectable liver only or liver predominant metastases (typically involving > 25% but < 60% of the liver, and technically inoperable, or unfeasible secondary to medical co-morbidity) 6. Have measurable disease by RECIST 1.1 criteria 7. Life expectancy of > 12 weeks 8. ECOG/WHO Performance Status of 0–1 9. Adequate liver function (bilirubin less than 34 umol/L in the absence of a reversible cause) 10. Blood work: patients must have °Platelet count of > or = to 50 × 10^9^/L °Hb of > 8.5 g/dL °ALT and AST < 5 x Upper limit of normal (ULN) °Serum creatinine < 1.5 x ULN °INR < 2.0 11. Patients with portal vein thrombosis may be considered, as determined at MDT	1. Clinically apparent ascites or other signs of hepatic failure on physical examination 2. Severe uncontrollable coagulopathy 3. No safe vascular access to the liver, as determined by triple phase CT 4. Potential for excess radiation exposure (>30Gy) to the lungs, as determined by pre-treatment 99mTc-MAA lung shunt (> 20% shunt) 5. Shunting to the GI tract that cannot be corrected by embolization, as demonstrated by hepatic angiogram 6. Previous TACE or SIRT 7. Multiple biliary stents, or ongoing cholangitis, or any intervention for, or compromise of, the Ampulla of Vater 8. Previous external bean radiotherapy to the liver 9. Systemic anti-cancer therapy within the last 4 weeks (excluding SSA) 10. Treatment with VEGF inhibitors within 3 months prior to therapy 11. Previous or concurrent cancer, other than Basal Cell Carcinoma, unless treated curatively 5 or more years prior to entry 12. Tumour involvement of > 60% of the liver 13. Oesophageal bleeding during the last 3 months 14. Any history of hepatic encephalopathy 15. Transjugular intrahepatic portosystemic shunt (TIPS) 16. Must not be at risk of hepatic or renal failure 17. Contraindications against angiography 18. Pregnancy and breast feeding. Women of child-bearing potential must have a negative pregnancy test 14 days before treatment, and at the time of TheraSphere administration. 19. Subjects with another significant medical, psychiatric, or surgical condition, currently uncontrolled by treatment, which may interfere with completion of the study. 20. Must not be participating in concurrent clinical trials evaluating treatment intervention(s).

### Study procedures

**Fig. 1:**
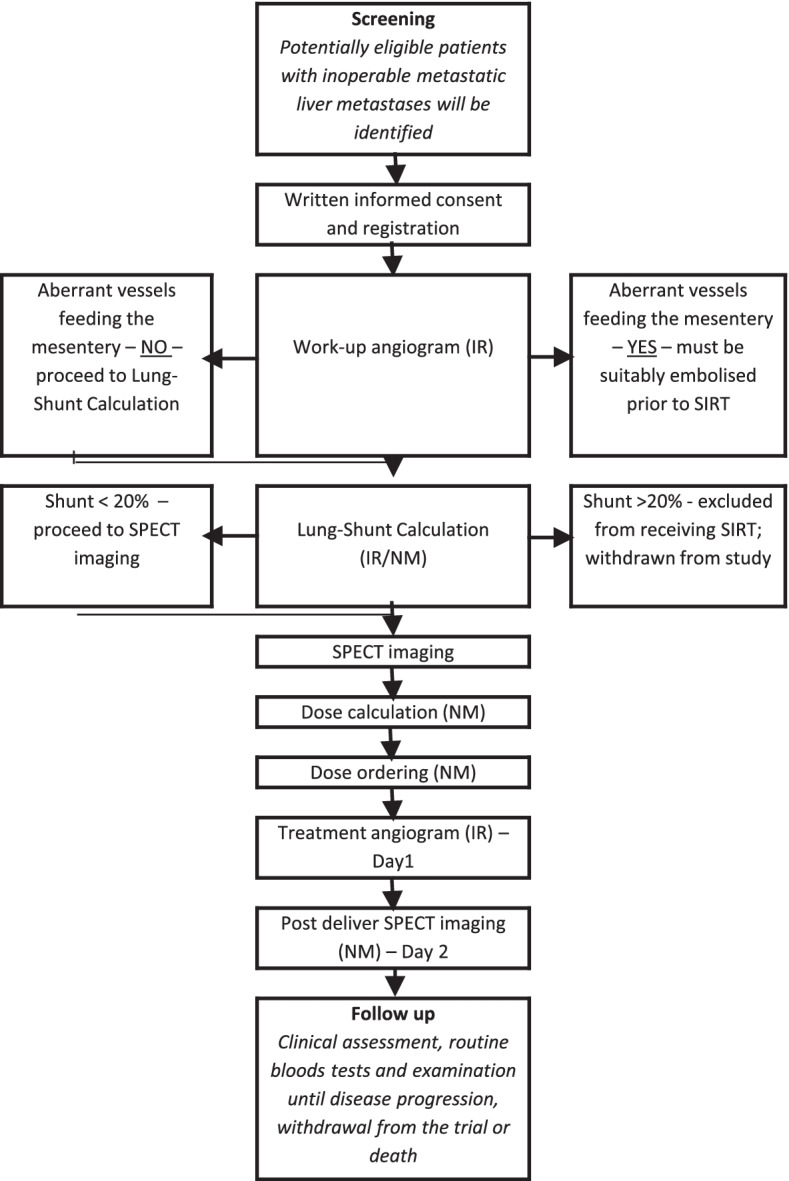
Trial Schema and TheraSphere Treatment Schedule

Patients will undergo baseline tumour imaging including CT scan of the chest, abdomen and pelvis, or magnetic resonance imaging (MRI) scan of the liver at screening (Fig. 1). Up to 4 weeks following consent, patients will undergo angiography of the hepatic artery to assess for the presence of aberrant vessels arising from hepatic arteries. If present, protective coiling of any extrahepatic branch (for example; aberrant segment IV and right gastric artery) will be undertaken. In the same session, technetium-99 (^99^Tc) macroaggregated albumin (MAA) will be injected into the hepatic artery via the same catheter position chosen for the scheduled SIRT session in order to calculate the hepatopulmonary shunt fraction and tracer distribution will be evaluated using ^99^Tc-MAA single-photon emission computed tomography (SPECT) imaging. Following completion of angiogram and ^99^Tc-MAA, a decision will be made regarding the dose of radiotherapy to be delivered. The activity and dose of SIRT will be calculated according to the body surface area model with the aim of delivering 120Gy of absorbed dose to the tumour. The administration of TheraSpheres will then take place up to 2 weeks following these investigations.

Patients will be admitted the night before treatment for a 24 hour octreotide infusion (100 mcg/ hour) in order to prevent carcinoid crisis. This will be continued for 24 hours post-treatment. SIRT will be conducted either as a lobar, sectoral, or segmental approach according to tumour size and location. In patients with bilobar disease, the first treatment will be administered to the lobe with the greatest tumour burden. Treatment of the contralateral lobe will be scheduled for 4–6 weeks after the first treatment. Following administration of TheraSpheres patients will receive standard of care post-TheraSphere treatment to prevent radiation-induced hepatitis and gastric complications: reducing dose of dexamethasone 8 mg daily for 1 week, 6 mg daily for the following week, 4 mg daily the week after that and 2 mg daily for one final week, proton pump inhibitor 60 mg daily and ursodeoxycholic acid 250 mg every night. A post-therapy SPECT will be performed 1 day after TheraSpheres are administered. Patients will be discharged the day after SIRT administration.

Patients will be reviewed week 4, 8, 12 and on a three-monthly basis until disease progression, withdrawal from the trial or death (whichever comes first). Blood tests will be performed at each visit to enable biomarker evaluation and safety. Tumour imaging will be repeated 3monthly post SIRT until tumour progression. The same method used for assessment at baseline must then be used at all subsequent time points. RECIST v1.1 criteria will be used to determine patient response to treatment, PFS and ORR. Participants will be assessed every 3 months thereafter to collect information regarding disease status and survival. QoL questionnaires (EORTC QLQ-C30) and EORTC QLQ-GINET21) will be completed at baseline, week 8 and 3monthly post SIRT until tumour progression.

### Safety

All adverse events (AEs) and adverse reactions (ARs) will be reported in a timely fashion. Adverse events and adverse reactions, whether expected or not, will be collected and recorded during clinical assessments at weeks 4, 8, 12 and 3-monthly thereafter as well as at the time of progression. When serious adverse events (SAEs) occur, an SAE form will be completed within 24 hours. The Chief Investigator will determine whether SAEs were ‘related’ (resulting from administration of the study treatment or procedure) or ‘unexpected,’ (an event not listed in the study protocol as an anticipated occurrence) and report this to the Research Ethics Committee. Any questions concerning adverse event reporting will be directed to the Chief Investigator in the first instance. The Chief Investigator will notify the Sponsor of all SAEs that occur. Potential related SAEs are included in supplementary data.

### Data collection

Data will be collected using trial specific patient report forms. As far as possible, missing data will be chased. For the primary analysis, there will be no data imputation for missing data in the primary end point.

### Translational endpoints

Alongside primary clinical endpoints, ArtiSaN will generate a biorepository of peripheral blood and imaging. Serum and plasma will be separated. Serum will be used to explore changes in circulating angiogenic factors using multiplex ELISA. cfDNA will be extracted from plasma and targeted sequencing undertaken. Imaging will be obtained and regions of interest manually drawn. Using established methodologies [13], we will extract radiomic features and correlate these with outcome measures using lasso regression.

### Statistical analysis

Assuming an ORR of 40%, a sample size of 24 patients, the 95% confidence interval will extend 20% in either direction. Three additional patients will be recruited to account for patient drop-out. Participants failing screening will not count towards recruitment Figs.

A full statistical analysis plan will be drawn up prior to database lock and any data interpretation. No interim analysis is scheduled to occur. The final analysis will be conducted at the end of the study once recruitment and treatment are complete.

The efficacy analysis will consist of the full analysis set (FAS), i.e., all patients who received SIRT. In the primary analysis, an estimation of ORR and its 95% confidence interval will be reported. If appropriate and required, a Per Protocol Analysis Set (PPS) will be defined/finalised/approved prior to final analysis on which the primary analysis will be repeated.

The safety analysis will also be conducted in the FAS. The frequency of ADEs and SADEs will be assessed for severity (NCI-CTCAE v4.03), expectedness, seriousness and causal relationship to treatment. ADEs will be summarised by toxicity, type and timing.

Histograms and box plots will be used to check the distribution and possible outliers for continuous variables. Continuous variables that follow a normal distribution will be summarised using means and standard deviations. Skewed continuous variables will be summarised using medians and inter-quartile ranges. Categorical variables will be summarised using frequencies and percentages.

Any deviation(s) from the final statistical plan in the final analysis will be described and justification given in the final report.

## Discussion

The presence of liver metastases in NENs is an independent prognostic factor and there has been significant interest in targeting liver metastases with a view to improve outcomes [3]. The current ESMO guidelines suggest that for the management of liver metastases, liver directed therapy can be considered the choice of which will depend on local expertise given the lack of randomised trials in this area [15], a position that makes clinical decision making difficult. Moreover, further questions arise as to when liver directed should be undertaken as opposed to systemic therapy. It is clear from the literature that systemic therapies including PRRT result in low objective response rates particularly in patients with bulky liver metastases and there may be role in liver directed therapy as an adjunctive measure.

The Hepar1 study attempts to address the issue of sequencing by investigating consolidative SIRT therapy following Lutathera in patients with liver metastases [1]. The study reported response rates up to 43% with acceptable side-effects profile with maintenance of quality of life outcomes following therapy prompting a phase III study to further investigate the role of liver consolidation. Further studies are proposed building on the combination of SIRT and systemic therapy including combination with capecitabine and temozolomide (NCT04339036 and NCT04789109) and immunotherapy (NCT03457948), the later phase II trial randomising patients to receive immunotherapy in combination with either PRRT or SIRT.

Recent real world registry data in NENs treated with SIRT reported survival figures of 33.1 months (95% CI: 22.1 – not reached) compared with of 16.5 months (95%CI: 14.2–19.3) for hepatocellular cancer and 9.8 months (95%CI: 8.3–12.9) for colorectal cancer, two tumour types were SIRT is recommended by international treatment guidelines [9]. This trial will add prospective clinical outcomes to a space dominated by retrospective work, giving further support for the use of SIRT in NENs. Moreover, we will generate much needed biomarker work for prediction of clinical outcome in this patient group including radiomics which may aid in treatment choice for patients moving forward.

## Supplementary Information

**Additional file 1.**

## Data Availability

Not applicable.

## Abbreviations

ADEs: Adverse device events
AEs: Adverse events
ARs: Adverse reactions
CT: Computed tomography
ENETs: European Neuroendocrine Tumour Society
EORTC: European Organisation for Research and Treatment of Cancer
FAS: Full analysis set
^166^Ho: Holmium-166
MAA: Macroaggregated albumin
MRI: Magnetic resonance imaging
NCI-CTC: National Cancer Institute - Common Toxicity Criteria
NENs: Neuroendocrine neoplasias
ORR: Objective response rate
OS: Overall survival
PRRT: Peptide related radiation therapy
PPS: Per Protocol Analysis Set
PFS: Progression free survival
QoL: Quality of life
SIRT: Selective Internal Radiation Therapy
SADEs: Serious adverse device events
SAEs: Serious adverse events
SSAs: Somatostatin analogues
SPECT: Single-photon emission computed tomography
^99^Tc: Technetium-99
TACE: Transarterial embolization with chemotherapy
TAE: Transarterial embolization
^90^Y: Yttrium-90
