# Supplementary material for: ArTisaN trial protocol: a single Centre, open-label, phase II trial of the safety and efficacy of TheraSphere selective internal radiation therapy (SIRT) in the treatment of inoperable metastatic (liver) neuroendocrine neoplasia (NENs)
Source: BMC Cancer. 2022 Jul 20;22:800. doi: 10.1186/s12885-022-09859-9 (PMC9297626; doi:10.1186/s12885-022-09859-9)
Supplement: Supplementary file 1 — Additional file 1. [file 12885_2022_9859_MOESM1_ESM.docx]

Allergic reaction, altered liver function, anorexia, anxiety, ascites, bile duct injury, bleeding/haemorrhage, chills/rigors, cholecystitis (inflammatory or infectious), colitis, death, dehydration, diarrhoea, dizziness, dyspnea, oedema (any
location), electrolyte abnormalities, elevated creatinine, falls, fatigue, fever, gastrointestinal bleeding/haemorrhage, gastrointestinal ulcer or ulceration, hepatic encephalopathy, hepatorenal failure, hiccups, hypertension, hypotension, infection (any location), liver failure, acute or chronic, lymphopaenia, malaise, mood alteration, muscle weakness, nausea, neutropenia, pain (any location), pancreatitis, platelet count abnormalities, pleural effusion, portal hypertension, pre-existing chronic liver disease decompensation, pulmonary oedema, pulmonary fibrosis, radiation hepatitis, radiation induced disease, radio embolization induced liver disease, sepsis, supraventricular arrhythmia, thrombosis (arterial or venous), tumour inflammation (including tumour oedema), tumour-lysis syndrome, vomiting, weight loss. Complications related to the administration procedure may include: allergic reaction, arterial injury including vessel dissection, aspiration pneumonia, bruising/bleeding/hematoma at site, constipation/abdominal distension, fatigue, flushing, infection, nausea, nerve damage^1^.

1. Ltd. BU. THERASPHERE™ Y-90 Glass Microspheres. 2021. <https://www.bostonscientific.com/content/dam/bostonscientific/pi/portfolio-group/cancer-therapies/therasphere/resource-center/product-info/TheraSphere_Fact_Sheet.pdf> (accessed 22/4/22 2022).
